# Comparing Outcomes of Community-Acquired Pneumonia Patients Discharged from General Medicine and Respiratory Units in Australia: A Propensity Score-Matched Analysis

**DOI:** 10.3390/jcm13103001

**Published:** 2024-05-20

**Authors:** Yogesh Sharma, Arduino A. Mangoni, Rashmi Shahi, Chris Horwood, Campbell Thompson

**Affiliations:** 1 College of Medicine & Public Health, Flinders University, Adelaide, SA 5042, Australia; arduino.mangoni@flinders.edu.au (A.A.M.); rashmi.shahi@flinders.edu.au (R.S.); 2 Division of Medicine, Cardiac & Critical Care, Flinders Medical Centre, Adelaide, SA 5042, Australia; chris.horwood@sa.gov.au; 3 Discipline of Medicine, The University of Adelaide, Adelaide, SA 5005, Australia; campbell.thompson@adelaide.edu.au

**Keywords:** community-acquired pneumonia, length of hospital stay, mortality, respiratory unit, general medicine unit, mortality, readmissions

## Abstract

**Background/Objectives**: Community-acquired pneumonia (CAP) is a common emergency presentation in Australia, with the choice of admitting specialty unit often influenced by pneumonia severity and comorbidities. However, it remains unclear whether there are between-specialty differences in patient characteristics and outcomes. We sought to address this issue by investigating the characteristics and outcomes of CAP patients admitted to General Medicine (GM) versus Respiratory units. **Methods**: This retrospective observational study utilised data from the two largest metropolitan hospitals in South Australia, encompassing all non-COVID-19-related CAP admissions throughout 2021 to 2023. The hospital length of stay (LOS), in-patient and 30-day mortality, and 30-day readmission rates were assessed by propensity score matching (PSM) using 17 variables. **Results**: Of the 3004 cases of non-COVID-19 CAP admitted across the two hospitals during the study period, 2673 (71.8%) were admitted under GM units and 331 (9.1%) under Respiratory units. GM patients were, on average, a decade older, presented with a significantly higher burden of comorbidities, exhibited a greater prevalence of frailty, and had higher pneumonia severity compared to those admitted under a Respiratory unit (*p* < 0.05). Unadjusted analysis revealed a shorter median LOS among GM-admitted patients (5.9 vs. 4.1 days, *p* < 0.001). After PSM adjustment, patients admitted under the Respiratory units had an 8-fold higher odds of a longer LOS compared to GM (adjusted odds ratio [aOR] 8.53, 95% CI 1.96–37.25, *p* = 0.004). Other clinical outcomes were comparable between the two groups. **Conclusions**: Our findings indicate that GM units compared to Respiratory units provide efficient and safe care for patients requiring hospitalisation for CAP.

## 1. Introduction

Community-acquired pneumonia (CAP) is a common presentation to emergency departments, often leading to hospitalisation for patients with moderate to severe illness. Research indicates that CAP contributes to approximately 2% of all emergency hospitalisations in Australia [1]. The in-hospital mortality rates from CAP range from 5% to 15%, escalating to as high as 30% among those necessitating admission to the intensive care unit (ICU) [2,3]. Furthermore, up to 17% of CAP patients may experience readmission within 30 days following hospital discharge [4]. Consequently, CAP imposes a substantial economic burden on hospitals. A UK study [5] reported that the mean costs associated with CAP hospitalisation were GBP 3904, with annual costs totalling GBP 731 million in 2019. The decision to admit patients with CAP typically rests with emergency department physicians, who weigh factors such as the severity of pneumonia, existing comorbidities, and resource availability to determine whether admission under General Medicine (GM) or Respiratory units is most appropriate [6].

Individual Australian hospitals have established guidelines for care of CAP patients, which prioritise admissions under Respiratory units for severe cases, aiming to optimise patient outcomes [7,8]. In general, frail older patients and those with complex comorbidity are more likely to be admitted under a GM unit than under a Respiratory unit [6]. However, real-world practice may deviate from these guidelines due to various factors such as presentation ambiguity, bed availability, or individual physician discretion, resulting in patients with severe illness also being admitted under GM units.

The clinical outcomes of CAP patients can vary significantly depending on multiple factors, including the severity of pneumonia [9]. Even after adjusting for these factors, studies have observed differences in clinical outcomes, such as the length of hospital stay (LOS), suggesting potential variations in the quality of care across different sites and between GM and specialist services within the same site [10,11].

Limited evidence exists regarding the characteristics and outcomes of CAP patients admitted under generalist versus respiratory care. Some studies have suggested comparable outcomes [6,12], while others [13] have indicated potential benefits, such as reduced mortality and shorter LOS, associated with admission to Respiratory units. These benefits may stem from expedited antibiotic initiation, the use of adjunctive therapies such as corticosteroids, and the early use of fibre optic bronchoscopy which can aid in early pathogen detection [13].

However, previous research [6,13] has not consistently accounted for other critical factors such as pneumonia severity [9] or frailty status [11], which are known to influence clinical outcomes among patients with CAP. Therefore, we sought to address this gap by examining the characteristics and outcomes of CAP patients discharged from two distinct specialist services: GM and Respiratory, considering the severity of illness and frailty status in the analysis. This approach should provide a more comprehensive understanding of the impact of different units’ specialist care on CAP outcomes, aiding in the refinement of clinical practices and resource allocation strategies.

## 2. Materials and Methods

This retrospective cohort study encompassed all emergency admissions for CAP at the two largest tertiary hospitals in Adelaide, South Australia—Flinders Medical Centre (FMC) and Royal Adelaide Hospital (RAH)—spanning from 1 January 2021 to 31 December 2023. CAP admissions were captured using the International Classification of Diseases, 10th Revision, Australian Modification (ICD-10-AM) diagnostic codes (J12-18.9) [14]. Patients with hospital-acquired pneumonia (HAP)—when pneumonia was acquired >48 h of hospital admission—as well as those who tested positive for severe acute respiratory syndrome coronavirus 2 (SARS-CoV-2) on viral polymerase chain reaction (PCR) were excluded. Ethical approval for this study was obtained from both the Southern Adelaide Human Clinical Research Ethics Committee (SA HREC) and the Central Adelaide Human Research Ethics Committee.

Patient and clinical data were retrieved from the Electronic Medical Records (EMR) of the two hospitals. Pneumonia severity was evaluated using the CURB-65 score [3] on admission, computed from parameters including confusion, urea concentrations > 7 mmol/L, respiratory rate > 30/min, blood pressure (systolic < 90 mmHg and/or diastolic < 65 mmHg), and age > 65 years. Frailty status was determined utilising the Hospital Frailty Risk Score (HFRS) [15], which uses a score ≥ 5 to classify patients as frail. Various comorbidities [16,17] which can influence outcomes among patients with CAP were identified, including chronic lung disease (chronic obstructive lung disease, (COPD), bronchial asthma, bronchiectasis, and interstitial lung disease (ILD)), coronary artery disease (CAD), chronic kidney disease (CKD), and history of cancer. Comorbidity burden was quantified using the Charlson Comorbidity Index (CCI) [18]. The nutritional status of patients was assessed using the Malnutrition Universal Screening Tool (MUST), which uses a cut-off score ≥ 1 to classify patients as malnourished [19]. Furthermore, routine blood investigations on admission, such as haemoglobin (measured in g/L; normal range: males 135–175 g/L and females 115–165 g/L), white blood cell (WBC) count (measured as no. of cells × 10^9^/L; normal range: 4.0–11.0 × 10^9^/L), the C-reactive protein (CRP) (measured as mg/L; normal range: <8 mg/L), albumin (measured in g/L; normal range: 34–48 g/L), creatinine (measured in μmol/L; normal range: males 60–110 μmol/L and females 45–90 μmol/L), and the international normalized ratio (INR), were extracted. Patients who had medical emergency response team (MET) calls and those who required admission to the ICU were determined. In addition, we retrieved data for patients who required high-flow oxygen therapy (HFOT) (defined as the need for 100% humidified oxygen at a flow rate of up to 60/L/min), non-invasive ventilation (NIV), mechanical ventilation, and vasopressor support during hospitalisation.

### 2.1. Outcomes

The outcome measures included the LOS adjusted for in-hospital deaths, in-hospital mortality, 30-day mortality from admission, and the 30-day readmission rate after discharge.

### 2.2. Statistical Analysis

Statistical analyses involved assessing variables for normality via histogram visualisation. Continuous variables underwent evaluation using *t*-tests or rank-sum tests, while categorical variables were analysed using Chi-square statistics.

#### 2.2.1. Propensity Score Methods

Propensity score matching (PSM) was used to mitigate the potential confounding variables between patients treated under GM vs. Respiratory units. A multivariable logistic regression model was developed based on confounding variables showing an association with a *p*-value of <0.20 in univariate analyses, with the patients receiving treatment under Respiratory units used as an exposure variable and hospital LOS as an outcome variable. Seventeen variables were included in this model, with matching performed at a 1:1 ratio on propensity scores utilising nearest neighbourhood matching [20]. These variables included the following: age, sex, CCI, CURB-65 scores, HFRS, MUST scores, haemoglobin, CRP, albumin, creatinine, the presence of chronic lung disease, cancer history, CAD, CKD, ICU admission, HFOT, and NIV. The balance of the characteristics between the cohorts was assessed using standardised mean differences (SMD), with SMD >10% considered as significant between the two groups [21]. In addition, propensity score distribution was visually inspected pre- and post-matching by plotting a distribution curve.

The differences in the outcomes between the two specialty units were assessed using the average treatment effect in the treated (ATET) with determination of odds ratios (OR) with corresponding 95% confidence intervals (CI).

#### 2.2.2. Sensitivity Analysis

We validated the PSM results using the inverse probability of treatment weighting (IPTW) [22], with ORs computed along with robust standard errors (SE) and corresponding 95% CIs. A significance threshold of *p* < 0.05 was applied for all tests, and Stata software version 18.0 was used for all statistical analyses (StataCorp LLC, College Station, TX, USA).

## 3. Results

### 3.1. Patient Characteristics

Over the three-year period, 3004 cases of non-COVID-19 CAP were admitted at the two hospitals. Of these, 2673 (71.8%) were managed under the GM units and 331 (9.1%) were treated under the Respiratory units. Patients admitted under GM were, on average, a decade older, presented with a significantly higher burden of comorbidities, and exhibited a greater prevalence of frailty (*p* < 0.05) compared to those admitted under Respiratory units (Table 1). Although pneumonia severity, as assessed by CURB-65 scores, was higher among GM-admitted patients, the Respiratory units managed a greater proportion of smokers and individuals with chronic lung disease (*p* < 0.05). Conversely, GM-admitted patients showed a higher prevalence of CAD and CKD (*p* value < 0.05). Notably, the patients managed by the Respiratory team had higher albumin concentrations and a greater likelihood of bacterial aetiology on culture results compared to those under GM care (*p* < 0.05) (Table 1). Patients admitted under Respiratory units also had a higher likelihood of admission to the ICU and were more likely to receive HFOT, NIV, and vasopressor support when compared to GM (*p* < 0.05) (Table 1).

### 3.2. Outcomes

#### Unadjusted Analysis

**Figure 1:**
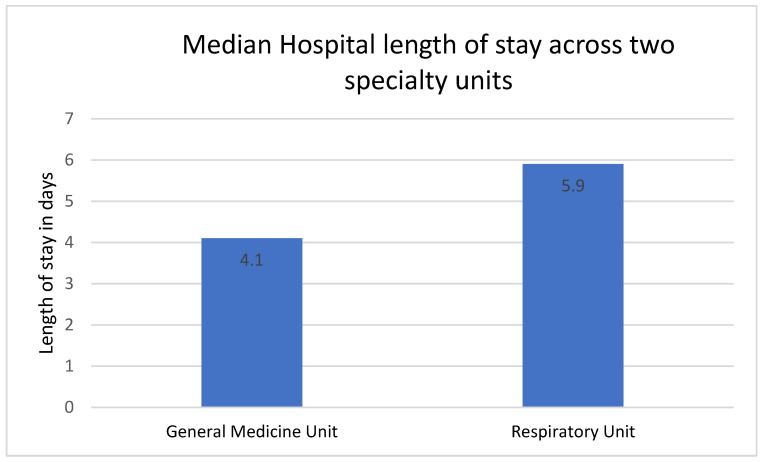
Comparison of median hospital length of stay across General Medicine and Respiratory units.

Unadjusted analysis revealed a 1.8 days shorter median LOS among GM-admitted patients (5.9 vs. 4.1 days, *p* < 0.001) (Table 2 and Figure 1). The in-hospital mortality rates were comparable, but 30-day mortality was significantly higher in the older and frailer GM cohort compared to the Respiratory unit cohort (13% vs. 9.1%, *p* = 0.041). Other outcomes were similar between the two groups (Table 2).

### 3.3. PSM

**Figure 2:**
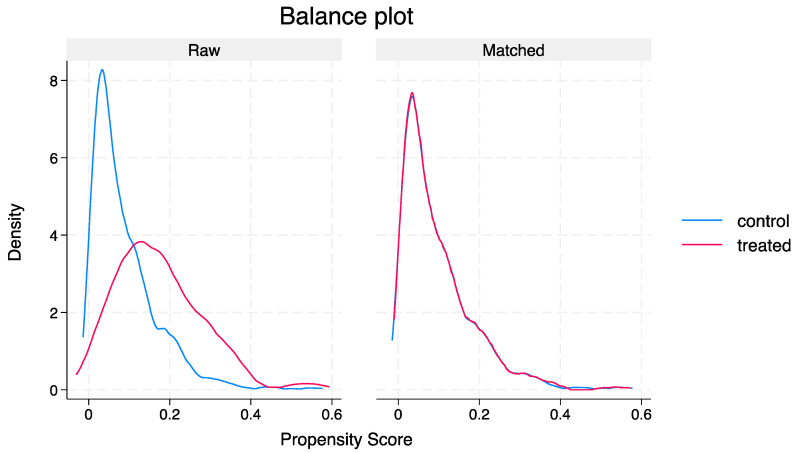
Distribution curve before and after propensity score matching.

Utilising 17 variables, PSM yielded 150 well-matched pairs with an SMD < 10% (Figure 2 and Table 3). An Assessment of Average Treatment Effect on the Treated (ATET) indicated that the patients managed by Respiratory units were more likely to experience a significantly longer LOS compared to GM (adjusted odds ratio [aOR] 8.53, 95% CI 1.96–37.25, *p* = 0.004). No significant differences were observed in in-hospital mortality (aOR 1.02, 95% CI 0.97–1.07, *p* = 0.775) or 30-day mortality (aOR 0.96, 95% CI 0.91–1.02, *p* = 0.101) between the two groups. Other clinical outcomes were also comparable (Table 4).

### 3.4. Sensitivity Analysis

Sensitivity analysis (Table 4) corroborated the aforementioned findings. The median LOS was significantly higher among patients managed by Respiratory units compared to GM (aOR 6.87, Robust SE 3.00, 95% CI 0.99–12.75, *p* = 022), while other clinical outcomes exhibited no substantial disparities between the two groups (Table 5).

## 4. Discussion

This study suggests that most of the patients presenting with non-COVID-19 CAP to the two largest South Australian metropolitan hospitals were admitted to a GM unit. Notably, the patients admitted under the GM service were generally older, had multiple comorbidities, and exhibited greater frailty and pneumonia severity compared to those admitted under the Respiratory service. PSM analysis revealed a significantly shorter hospital LOS for CAP patients admitted under GM compared to those admitted under Respiratory; however, other clinical outcomes were similar between the two units.

The in-hospital mortality and readmission rates are regarded as better indicators of the quality of in-hospital care while LOS is more likely reflective of the efficiency of care [6]. The current study indicates that the LOS was 1.8 days shorter for patients who were admitted under GM when compared to the respiratory team. Our findings align with a previous Australian study [6] conducted in the pre-COVID-19 era, which reported a median LOS 1.5 days shorter under GM than Respiratory for 9775 emergency CAP hospitalisations over 5 years. Importantly, our study extends this knowledge by adjusting for pneumonia severity and frailty status, which are significant determinants of in-hospital LOS [23,24]. The significantly shorter LOS under GM in our study could potentially be attributed to the enhanced utilisation of allied health services, focusing on early mobilisation and efficient hospital-at-home services [25,26].

Previous research has demonstrated that complications during hospitalisation and ICU admissions are associated with an extended LOS in CAP patients [23]. The increased frequency of ICU admissions among patients under the Respiratory unit compared to GM in this study (21.7% vs. 5.8%, *p* < 0.001) may have also contributed to the longer LOS observed in this group.

Our findings suggest that the care provided to non-COVID-19 CAP patients admitted under GM units was more efficient compared to those admitted under Respiratory units. Consistent with previous studies [6,12], our results indicate that there was no compromise in the quality of care for GM patients, as evidenced by comparable in-hospital mortality and 30-day readmission rates between the two units. Prior research has demonstrated that a shorter LOS for CAP patients is associated with reduced healthcare costs [27]. Additionally, more patients are likely to return to their usual activities or work within 14 days following hospital discharge when LOS is minimised [28]. Therefore, the observed efficiency in GM units may not only improve patient outcomes but also contribute to cost savings for the health service and a faster return to functional status for the patient.

Contrary to our findings, several studies have reported better clinical outcomes for patients admitted under specialty services compared to generalist care. For instance, a prospective observational study by Pothirat et al. [29] involving 208 patients hospitalised with an acute exacerbation of COPD found that respiratory physicians were more likely to adhere to guideline-directed therapy. Patients under their care experienced fewer severe adverse events, had a shorter LOS, and incurred lower costs compared to those under generalist care (*p* < 0.05). However, no significant difference in in-hospital mortality was observed between the groups. Similarly, a study by Jong et al. [30] focusing on hospitalised heart failure patients revealed that those treated by cardiologists had a lower one-year risk of death (28.5% vs. 31.5%, *p* < 0.05) and composite risk of death and readmission (54.7% vs. 58.1%, *p* < 0.05) compared to non-cardiologists.

A major limitation of these earlier studies is the lack of adjustment for frailty and illness severity, significant determinants of clinical outcomes in hospitalised patients [31]. Additionally, previous studies did not utilise robust statistical procedures such as PSM. Moreover, the term “specialty care” was not clearly defined, contrasting this specialty care with that offered by general practitioners (GPs) or family physicians who may have different qualifications and discharge planning skills compared to general internal medicine physicians similar to those represented in the present study [32].

### Limitations

Our study has several limitations that warrant consideration. Firstly, we were unable to ascertain the type and duration of antimicrobial treatment for CAP or determine any differences in adherence to guideline-concordant antibiotic treatment between the two specialties. Secondly, the exclusion of COVID-19-positive patients limits our ability to quantify the influence of the COVID-19 pandemic on CAP outcomes. Lastly, this was not a study where patients were prospectively randomised to either the Respiratory or the GM units. Therefore, the inherent biases present in the admission processes at either or both hospitals (beyond age, comorbidity, frailty, etc.) cannot be excluded.

## 5. Conclusions

In conclusion, our study suggests that the majority of hospitalised CAP patients are admitted under GM care, and those admitted under this care model, despite being older and more frail, had a shorter LOS without significant differences in other clinical outcomes. However, there is a need for future prospective clinical studies to validate our findings and explore the potential benefits and limitations of different care models in the management of CAP.

## Figures and Tables

**Table 1:** Characteristics of non-COVID-19 community-acquired pneumonia patients admitted under two specialties.

	Total	General Medicine	Respiratory	*p* Value
No. (%)	3004	2673 (71.8)	331 (9.1)	
Age years, mean (SD)	73.4 (17.4)	74.5 (17.1)	64.2 (17.6)	<0.05
Sex male *n* (%)	1646 (54.8)	1454 (54.4)	192 (58.0)	0.213
CCI mean (SD)	2.3 (2.7)	2.4 (2.8)	1.9 (2.3)	<0.05
CURB-65 mean (SD)	1.8 (1.1)	1.8 (1.1)	1.4 (1.1)	<0.05
HFRS mean (SD)	5.4 (4.8)	5.5 (4.9)	4.0 (3.8)	<0.05
MUST score mean (SD)	0.6 (1.1)	0.7 (1.1)	0.6 (1.2)	0.872
Chronic lung disease *n* (%)	1109 (36.9)	943 (35.3)	166 (50.2)	<0.05
CAD *n* (%)	269 (8.9)	250 (9.4)	19 (5.7)	<0.05
CKD *n* (%)	427 (14.2)	404 (15.1)	23 (6.9)	<0.05
Cancer *n* (%)	354 (11.8)	304 (11.4)	50 (15.1)	0.05
Smokers *n* (%)	145 (4.8)	114 (4.3)	31 (9.4)	<0.05
Alcohol *n* (%)	165 (5.5)	148 (5.5)	17 (5.1)	0.763
Positive bacterial culture *n* (%)	326 (10.9)	247 (9.2)	79 (23.9)	<0.05
Haemoglobin g/L mean (SD)	120.6 (20.4)	120.5 (20.5)	120.9 (19.9)	0.739
WBC × 10^9^/L mean (SD)	12.8 (7.5)	12.8 (7.5)	12.9 (6.9)	0.816
CRP mg/L mean (SD)	109.5 (104.3)	108.4 (102.4)	118.8 (119.1)	0.096
Creatinine mmol/L mean (SD)	102.8 (75.3)	104.7 (77.8)	87.2 (47.7)	<0.05
Albumin g/L mean (SD)	29.6 (5.5)	29.7 (5.5)	28.5 (5.8)	<0.05
INR mean (SD)	1.4 (0.7)	1.4 (0.7)	1.3 (0.6)	0.352
MET calls *n* (%)	432 (14.4)	376 (14.1)	56 (16.9)	0.163
ICU admission *n* (%)	138 (4.6)	94 (3.5)	44 (13.3)	<0.05
ICU Length of stay in hrs median IQR	74.5 (44, 140.5)	75 (41, 134)	75.5 (53, 148.5)	0.394
High-flow oxygen *n* (%)	68 (2.3)	41 (1.5)	27 (8.2)	<0.05
NIV *n* (%)	33 (1.1)	13 (0.5)	20 (6.0)	<0.05
Mechanical ventilation *n* (%)	14 (0.5)	13 (0.5)	1 (0.3)	0.642
Vasopressor support *n* (%)	113 (3.8)	90 (3.4)	23 (6.9)	<0.05

All continuous variables were compared using the *t*-tests except ICU length of stay, which was assessed by use of the rank-sum test, and categorical variables were compared using the Chi-square statistic; COVID-19, coronavirus disease; SD, standard deviation; CCI, Charlson Comorbidity Index; CURB65, (pneumonia severity score calculated from following parameters: confusion, urea levels > 7 mmol/L, respiratory rate ≥ 30/min, blood pressure systolic < 90 mm Hg or diastolic < 60mm Hg, and age ≥ 65 years); HFRS, hospital frailty risk score MUST, Malnutrition Universal Screening Tool; CAD, coronary artery disease; CKD, chronic kidney disease; WBC, white cell count; CRP, C-reactive protein; INR, international normalised ration; MET, medical emergency response team; ICU, intensive care unit; NIV, non-invasive ventilation.

**Table 2:** Outcomes of non-COVID-19 community-acquired pneumonia patients admitted under two specialty units.

Outcome	Overall	General Medicine	Respiratory	*p* Value
No. (%)	3004	2673 (78.9)	331 (11.1)	
Length of stay, median IQR	3.9 (2, 7)	4.1 (2.5, 7.1)	5.9 (3.5, 9)	<0.05
In-hospital mortality *n* (%)	215 (7.2)	199 (7.4)	16 (4.8)	0.082
30-day mortality *n* (%)	378 (12.6)	348 (13.0)	30 (9.1)	<0.05
30-day readmissions median IQR	481 (16.0)	428 (16.0)	53 (16.1)	0.100

Length of stay compared by use of the rank-sum test and categorical variables compared by use of the Chi-square statistic; COVID-19, coronavirus disease; IQR, interquartile range.

**Table 3:** Standardised mean differences and variance ratios in raw and matched pairs.

Standardised Mean Differences			Variance Ratio	
Variable	Raw	Matched	Raw	Matched
Age	−0.56	−0.09	1.11	0.88
Sex	0.05	−0.08	0.99	1.01
CCI	−0.22	0.76	0.75	1.28
CURB-65	−0.54	0.01	0.86	0.96
MUST_score	−0.04	−0.06	1.01	0.84
HFRS	−0.53	−0.03	0.47	0.69
Haemoglobin	0.07	−0.06	0.99	1.26
CRP	−0.12	0.10	0.97	1.52
Creatinine	−0.31	0.08	0.03	0.58
Albumin	−0.11	0.01	1.35	1.71
Chronic lung disease	0.35	0.09	1.07	0.94
Cancer	0.02	0.04	1.07	1.10
CAD	−0.11	−0.08	0.70	0.54
CKD	−0.26	−0.03	0.51	0.94
ICU admission	0.04	0.09	0.04	0.09
High-flow oxygen	0.01	0.05	0.01	0.04
NIV	0.01	0.06	0.01	0.07

CCI, Charlson Comorbidity Index; CURB-65, calculated from the following: confusion, urea > 7 mmol/L, respiratory rate > 30/min, blood pressure > 90 mmHg or diastolic < 65 mmHg, age > 65 years; MUST, Malnutrition Universal Screening Tool; CRP, C-reactive protein; CAD, coronary artery disease; CKD, chronic kidney disease; ICU, intensive care unit; NIV, non-invasive ventilation.

**Table 4:** CAP outcomes if admitted under Respiratory team compared to General Medicine after propensity score matching.

Outcome	Odds Ratio *	95% CI	*p* Value
LOS	8.53	1.96–37.25	0.004
In-hospital mortality	1.02	0.97–1.07	0.775
30-day mortality	0.96	0.91–1.02	0.101
30-day readmissions	0.95	0.87–1.05	0.336

* Odds ratio displaying average treatment effect on treated (ATET); CI, confidence interval.

**Table 5:** CAP outcomes if admitted under Respiratory team compared to General Medicine after inverse probability weighting.

Outcome	Odds Ratio *	Robust SE	95% CI	*p* Value
LOS	6.87	3.00	0.99–12.75	0.022
In-hospital mortality	1.01	0.02	0.98–1.05	0.461
30-day mortality	0.98	0.01	0.96–1.07	0.191
30-day readmissions	0.89	0.08	0.76–1.05	0.177

* Odds ratio displaying average treatment effect on treated (ATET); SE, standard error; CI, confidence interval.

## Data Availability

Data for this study can be obtained from the corresponding author upon reasonable request, subject to approval by the ethics committee.
